# Retraction Note: SNHG6 modulates oxidized low-density lipoprotein-induced endothelial cells injury through miR-135a-5p/ROCK in atherosclerosis

**DOI:** 10.1186/s13578-023-01093-5

**Published:** 2023-08-07

**Authors:** Haiyan Shan, Dawei Guo, Siyang Zhang, Huimeng Qi, Shen Liu, Yanmei Du, Yini He, Bofu Wang, Ming Xu, Xiaosong Yu

**Affiliations:** 1 Department of General Practice, The First Affiliated Hospital of China Medical University, No. 155 Nanjing North Street, Heping District, Shenyang, 110001 China; 2 Department of the Fourth General Surgery, The Fourth Affiliated Hospital of China Medical University, No. 4 Chongshan East Road, Huanggu District, Shenyang, 110032 China; 3 Department of the Science and Experiment Center, The China Medical University, No. 77 Puhe Road, Shenbei New Area, Shenyang, 110122 China

**Retraction Note: Cell & Bioscience (2020) 10:4**

10.1186/s13578-019-0371-2

The Editor-in-Chief has retracted this article after the investigation by the publisher found that potential image overlap in Figs. 2f, 4e, 5f, 6e and 7a. The authors failed to provide raw images and an ethical approval document.

In addition, the corresponding author stated that he was not aware of the publication of this article and his email address provided on submission was not his email address. None of the authors responded to any correspondence from the editor/publisher about this retraction.

